# Operationalising multi-sectoral food- and nutrition-related policies to curb the rise in obesity in Ghana

**DOI:** 10.1017/S136898002300037X

**Published:** 2023-12

**Authors:** Samuel Akwei Sackar, Charles Apprey, Linda Nana Esi Aduku, Anne Marie Thow, Reginald Annan

**Affiliations:** 1 Department of Dietetics, School of Biomedical and Allied Health Sciences, College of Health Sciences, University of Ghana, P.O. Box KB143, Accra, Ghana; 2 Department of Biochemistry and Biotechnology, Kwame Nkrumah University of Science and Technology, Kumasi, Ghana; 3 College of Science, Kwame Nkrumah University of Science and Technology, Kumasi, Ghana; 4 Menzies Centre for Health Policy, School of Public Health, University of Sydney, Sydney, NSW, Australia

**Keywords:** Multi-sectoral, Obesity, Operationalising, Power, Nutrition policies

## Abstract

**Objective:**

To examine the governance of the food and nutrition policy space with particular reference to interests and power among stakeholders.

**Design:**

We followed a case study research design to conduct a nutrition policy analysis. We triangulated three sources of data: key-informant interviews, learning journey and relevant policy documents (2010–2020). This study is grounded in a conceptual framework focused on power.

**Setting:**

Ghana.

**Participants:**

Key informants (*n* 28) drawn from policy stakeholders from government (Health, Agriculture, Trade and Industry), academia, civil society, development partners, civil society organisation (CSO) and private sector in Accra and Kumasi.

**Results:**

Power relations generated tensions, leading to weak multi-sectoral coordination among actors within the nutrition policy space. Governance and funding issues were identified as reasons for the weak multi-sectoral coordination. Formal power rested with government institutions while the private sector and CSO pushed to be invited during policy formulation. Visible stakeholders from industry were trade oriented and held a common interest of profit-making; they sought to receive support from government in order to be more competitive. There were no observed structures at the subnational levels for effective link with the national level.

**Conclusion:**

Formal responsibility for decision making within the nutrition and food policy space rested with the health sector and bringing on board nutrition-related sectors remained a challenge due to power tensions. Establishing a National Nutrition Council, with structures at the subnational level, will strengthen policy coordination and implementation. Taxation of sugar-sweetened beverages could provide a fund generation avenue for coordination of programmes to curb obesity.

Nutrition issues such as obesity have been on the increase in recent decades, and changes in food systems and environment have been seen to drive this increase^(1–3)^. In recent years, many African cities have experienced a massive shift from traditional informal markets towards modern retail centres such as supermarkets, restaurants and fast-food outlets^(4,5)^ which are linked with increased consumption of ultra-processed, less healthy foods^(4)^. For example, in their 2019 study, Khonje & Qaim identified a positive correlation between the increase in consumption of highly processed foods and the increasing rates of obesity and overweight in Zambia^(4)^.

This trend has also been reported in Ghana, as the streets of urban centres are well supplied with convenience stores, food vendors and hawkers who mostly trade in high calories foods^(6)^. In addition, several scholars have reported that fruit and vegetables are not readily accessible in some parts of Ghana – and where they are available, the cost is a restrictive factor, especially of fruit, thus limiting their consumption^(7–9)^.

The multi-sectoral approach has been recognised as one of the ways of addressing the multifaceted issues of nutrition, as it employs the efforts of various stakeholders with shared interests in a common target^(10–12)^. This approach is not new to Ghana. It was employed in the fight against HIV/AIDS, where the National AIDS Commission was set up to coordinate the activities of stakeholders from several sectors including health, local government and finance. Again, the problems of illegal mining also saw the use of a multi-sectoral approach, where an Inter-Ministerial Committee on Illegal Mining^(13)^ was set up to coordinate efforts in curbing illegal mining.

Tackling the intricate nature of obesity in this way brings together stakeholders within the nutrition policy space to deliberate on the best interventions required, leading to shared responsibilities to improve nutritional outcomes^(10,14,15)^. A comprehensive multi-sectoral approach involves whole-of-government approach, spanning sectors such as health, food and agriculture, finance, trade and industry, education and communication – and including relevant civil society organisations (CSO) and private entities.

A review of governmental agency reports and policy documents suggests that there has been some level of collaborations among stakeholders from agriculture, local government, academia, health and trade^(16–18)^ but lacks structures, especially at the subnational level for effective coordination. Thus, there have been increased calls for governance structures at both the national and subnational levels to ensure better stakeholder engagement and coordination for effective operationalisation of nutrition policies^(11,19,20)^.

Food system policy actors roles, mandates and interests in Ghana have been studied previously^(21,22)^, but there has been little explicit examination of power. This study thus extends previous research, using a power-focused analytical framework which has been used elsewhere to analyse nutrition policy^(23,24)^.

## Methods

This study is embedded within the Researching the Obesogenic Food Environment, potential policy levers in South Africa and Ghana (ROFE) study. The ROFE study, implemented in three phases, sought to better understand the changing food environment in South Africa and Ghana, drivers of this change, including the value chain actors within this environment, and potential policy levers and opportunities to improve the food environment. The objective of this study, embedded in the third phase of ROFE, was to examine the governance of the food and nutrition policy space, including interrogating policies related to obesity. The primary research questions were who are the key stakeholders in the nutrition policy space and what are their interests, what is the current state of the coordination and what is the governance and power structure and relations within the nutrition policy space?

Our analytical focus was the power relations among various stakeholders in the operationalisation of the multi-sectoral response to nutrition issues, with particular interest in addressing overweight and obesity in Ghana.

### Study design

We followed a case study research design^(25)^ to conduct a nutrition policy analysis. We triangulated three sources of data: key-informant interviews, learning journey and relevant policy documents. This study utilised qualitative research methods to examine the multi-sectoral governance of the food and nutrition policy space in Ghana. The study population was made up of participants from key regulatory and policy bodies, and stakeholders involved in national and local level food system and nutrition policy and governance.

### Analysis framework

This study is grounded in the framework of power developed by Gaventa (Table 1)^(26)^.

**Table 1:** Key terms relevant to power (Gaventa’s power cube)

Spaces	Levels	Forms
Closed: limited to a select few and usually controlled by the elites (elected officials or representatives, technocrats, bureaucrats) who render services or make decisions without the need for broader involvement or consultation	Global: where global forces determine the actors and agenda for discussion	Visible: the overt and often defined aspects of power exercised by formalised bodies like governmental agencies and ministries
Invited: expands the boundaries of closed space to involve others who were not initially part of the space as a result of several calls and pressures by the ‘outsiders’ (those who do not belong to the closed spaces)	National: power that usually resides in the capital cities and drives who and what is put on the table for discussion	Invisible: covertly shapes the perceptions of people by placing ideological and psychological boundaries on participation
Claimed: comes about when there is mobilisation around a common course or identity by less powerful actors feeling neglected by the power wielders who do not attend to their concerns	Local: this level has power residing at the grassroots, where more often, participation is opened to the local people	Hidden: they may be viewed as the controllers of those with the visible power and usually work to exclude representation and issues put up by other groups seen as less powerful

Gaventa’s power cube provides a basis to visualise power in terms of actors’ relationships and to identify strategies that could bring about changes in power relations and arrangements.

The first dimension is the spaces in which power is exercised. Spaces are not limitless but are regulated by boundaries which shape what is possible within them as well as who enters and with what interests and the discourses that occur within^(26,27)^. Thus, we identified the spaces within which actors operate and the availability of these spaces, especially to the private sector and CSO, who, usually, are seen as less powerful actors.

In Gaventa’s second dimension of power, the dynamics in the changing levels of power creates challenges for civil society as to where best to engage their efforts. While some dedicate their efforts on advocacy at the global level, others focus on local situations. However, concerns arise as to a disconnect in vertical links among actors within the levels. We therefore looked at the power relations at the national and subnational levels.

The third dimension, captured as forms of power, is closely related to Lukes’ three faces of power^(28)^. The forms include visible power (formal structures, institutions, authorities, rules and decision-making processes), invisible power (ideological and psychological boundaries on participation) and hidden power (powerful institutions and people who retain decision-making influence silently)^(26)^. Based on this, we captured the institutions that had formal responsibility for nutrition and assessed the existence of formal structures to enhance multi-sectoral coordination.

We therefore framed our analysis based on actors’ engagement in policy formulation and implementation, who really drives the policy agenda and the governance structure around the nutrition policy space.

### Data collection

A total of twenty-eight key informants were recruited from the two biggest economic cities in Ghana: Accra (national capital) and Kumasi (a regional capital) in January 2019 (Table 2). We identified potential interviewees from key regulatory and decision-making bodies such as the ministries of health, local government, agriculture and trade and industry, as well as from food-related CSO involved in national and local level food system policy and governance. Using the snowballing technique, additional key informants were identified until descriptive saturation was achieved^(29)^. Recruitment was through formal letters of invitation to heads of agencies, followed up with emails/phone calls, to identify appropriate participants. Interviews were tailored to the expertise of the participants and designed to understand the current framework for coordination and collaboration in nutrition policy formulation and implementation, institutional structures, actor interests, exercise of power and opportunities for multi-sectoral collaborations. We also asked participants about opportunities and points of leverage for policy change, to strengthen food policy decisions in terms of consideration of nutrition.

**Table 2:** Summary of characteristics of interviewees†

Type of organisation (*n* * = 26)	Location (*n* * = 26)	Main sectoral interest (*n* * = 26)
Academia (6)	Accra (3) Kumasi (3)	Public Health (2) Agriculture/Agribusiness (2) Nutrition/Food Science (2)
NGO/CSO (3)	Accra (3)	Industry (2) Agriculture (1)
National (14) and subnational (5) government	Accra (14) Kumasi (5)	Development (2) Agriculture (6) Industry/Trade (2) Health (3) Research (4) Regulatory (2)

CSO, civil service organisation; NGO, non-governmental organisation.

* *n* represents the number of persons interviewed.

† Interviewees are described at a high level to avoid identification.

The interviews in Accra targeted national-level governmental institutions while those in Kumasi occurred at the subnational levels. The interviews were conducted at the offices of the key informants and lasted 26–40 min. The interviews were recorded using an audio recorder and transcribed in full. In two cases, we were not granted permission to make audio recordings. As such, detailed notes were taken and written up afterwards.

The second type of data was collected during a 2-d learning journey and consultative workshop which took place in Kumasi in April 2019. A learning journey is a qualitative means of data collection where participants are taken to the natural setting under investigation to observe at first-hand what was happening^(30)^, in order to make informed inferences. The learning journey included visits to food systems in parts of Kumasi, while the participants took notice of the food environment as we drove to the locations. The sites visited were a local poultry farm (large-scale producers of poultry and poultry products), a distribution centre (distributors of processed foods) and the Bantama Market (an indigenous open market mostly for organic foods). These sites were chosen among others through balloting after which phone calls were made to the selected sites to seek permission. The twenty-eight key informants interviewed in the first part of data collection were invited to participate in the learning journey, out of which twenty-three of them or their representatives who were available participated. The consultative workshop took place a day after the learning journey on the Kwame Nkrumah University of Science and Technology (KNUST) campus. A representative from The Southern Africa Food Lab partnered with the KNUST team to facilitate the whole process.

The participants were put into groups of four to reflect on the learning journey and discuss their experiences.

Among others, these questions were used as a guide:

- What were the key highlights for you?
- What are some of the current realities of the food systems/environment in relation to obesity?
- How does the food systems/environment facilitate/hinder the access to healthy foods?
- How does the food systems/environment vary by other cities (local /foreign) you are familiar with?
- How can the observed issues be addressed multi-sectorally?

After 45 min of group discussions, each group documented its answers on sheets of papers provided and summarised their findings in a PowerPoint presentation. We collated the findings from each group and imported them into the ATLAS.ti software (version 7) for coding and analysis using our analytical framework as a guide.

Third, we identified relevant policy documents within the past decade by requests to key informants as well as through internet searches on google scholar and websites of governmental institutions such as the ministries of Health, Food and Agriculture, Trade and Industry and the Food and Drugs Authority. These documents included the National Nutrition Policy of Ghana, National Policy for the Prevention and Control of Chronic Non-Communicable Diseases in Ghana, the Planting for Food and Jobs policy, Health Sector Medium Term Development Plan, Ghana Shared Growth and Development Agenda II, the Scaling-Up Nutrition Movement website reports, online newspaper reports and public commentaries.

An excel sheet was designed to extract relevant data from the documents. Some dimensions used for the extraction included: the stated objectives of the policy, funding sources, whether nutrition is mentioned at all in the document, who to coordinate the implementation of the policy and the most important sectors mentioned in the document.

### Data analysis

The interviews data were transcribed using Microsoft Word 2016. The transcribed documents together with the data generated from the consultative workshop were then imported into ATLAS.ti version 7, a computer-assisted qualitative analysis software, for analysis. Based on the study framework, the data were coded into major thematic pre-determined codes, augmented with open coding, including stakeholder; engagement; institutional relationships; priority for nutrition; policy instruments; objectives/aims; coordination; consultation and collaboration; actor interests and policy priorities (Table 3). Drawing on documentary data from archival documents and content analysis of policy documents within the past decade, the data from the interview and learning journey were triangulated in conformity with case study research^(31)^.

**Table 3:** Coding framework

Pre-determined codes	Open coding
Stakeholder engagement, institutional relationships, priority for nutrition, policy instruments, objectives/aims, policy coordination, responsibility for coordination, governance structure, national institutional structures, subnational institutional structures	Actors’ interests, policy priorities, policy instruments, funding sources

## Results

### Overview

The Ministry of Agriculture, Ministry of Health (MOH) and Ministry of Trade and Industry were the main governmental institutions with formal responsibility relevant to nutrition and food policy. Within the nutrition policy space, leadership was provided by the MOH, through the Nutrition Unit of the Ghana Health Service. Power relations usually generated tensions both among governmental institutions and between government and the private sector and CSO. The government institutions usually demonstrated formal power while the private sector and CSO pushed to be invited during policy formulation and implementation. Visible stakeholders from industry were trade oriented and held a common interest of profit-making; they sought to receive support from government in order to be more competitive. In contrast, producers of healthy food were not perceived as influential in food policy. The data also indicated a weak multi-sectoral coordination among diverse sectors involved in the nutrition policy space. Issues with governance and funding, as well as tensions among the stakeholders, were identified as part of the reasons for the weak coordination of the various sectors.

### Key stakeholders and their interests

Among the several stakeholders identified within the nutrition and food policy space, government was recognised as the main stakeholder, possessing formal authority for decision making (visible power) and acting through its ministries and agencies such as health, agriculture, trade and industry. All other stakeholders were identified as supporting the efforts of government. Other identified stakeholders with interests in this policy space were industry, agricultural input dealers, food processors, farmers, traditional authorities, CSO and development partners. These actors, external to government, consistently sought more opened spaces for participation in policy formulation and implementation related to food and nutrition.

At the national level, international organisations such as the WHO, Alliance for Green Revolution in Africa, United States Agency for International Development and the World Food Programme (WFP) were identified as influencing government ministries and agencies in agenda setting and policy formulation. As noted by some of the interviewees, the interest of these international organisations was for the country to meet internationally agreed targets like the sustainable development goals. An example of such influence by international organisations (as discussed by some of the interviewees) was seen in the development of the National Nutrition Policy, which was in response to meeting the targets for the sustainable development goals set by the United Nations.

Some of the interviewees indicated that the interests of ministries such as agriculture and industry related to nutrition were directed towards food security but not nutrition security.

‘There is a misconception that by producing and increasing access to more foods without much thought to the nutritional quality of such foods, nutrition security will be ensured. And so, government focuses mainly on planting, planting, planting’. (Key informants – academia and national local government institution)

This was evident in the ‘planting for food and jobs’ document, which had specific targets to increase food production but no identified targets for nutrition. Similarly, nutrition was not an objective in the National Medium-Term Development Policy Framework of the Ministry of Agriculture. As mentioned by key informants from academia and health, the role of the Ministry of Trade and industry was observed to be dichotomous, on the one hand helping to increase availability of foods to ensure food security, while on the other hand, promoting the influx of high calorie but low nutrient foods which are precursors for obesity and overweight, as they sought to promote trade and industrial activities.

Data generated from the learning journey indicated that the interests of the food industry in the policy space were largely those related to trade, in particular livestock, sugar-sweetened beverages (SSB) and processed foods. In contrast, producers of unprocessed products like traditional foods, fruits and vegetables were not seen as being influential within the policy space. However, interests within the food industry were mainly driven by high profit margins without recourse to health and good nutrition and as such these industries pushed for policies that helped them become more competitive as stated by some of the interviewees. In particular, key informants from the private industries involved in agriculture noted that they were interested in government’s provision of the needed support for domestic industry (loans, infrastructure, storage, etc.) as well as technology to help with reducing post-harvest losses. As an example, data from the learning journey and interviews indicated that stakeholders within the poultry industry were mainly interested in the importation of frozen poultry meat. Some of the interviewees suggested that though this industry was positioned to help improve the protein intake of individuals by increasing access to poultry meat, the interests of importers were rather solely profit-driven, with no considerations to the high fat content of these imported frozen poultries. They also noted that the imported poultry meat was relatively cheaper than the locally produced ones (usually with less fat content) and, thus, were easily marketable and more profitable.

‘Some people who are into the poultry industry … are looking solely at profits. These ones are not looking at the nutrition aspect or quality, so once they see that it’s more profitable to import … they will keep bringing them in. And unfortunately for us most people buying from the market are not interested in the source, whether it is locally produced or imported, they are only interested in the cost; the cheaper the better’. (Key informant – Regional Public Health unit)

### State of multi-sectoral nutrition coordination in Ghana

Data from the interviews and documents analysis indicated some collaborations among the different stakeholder types, for example, between local government ministry and Ministry of Food and Agriculture regarding the implementation of food policies at the subnational level. However, as noted by a key informant from the local government ministry, there were issues of role misunderstanding and autonomy which usually generated tensions between the two ministries. Some key informants also noted little or no existing coordination between the nutrition unit of the MOH and other government sectors such as agriculture, water and sanitation and trade and industry when formulating nutrition-related policies.

Some of the interviewees noted that coordination processes always halted with a change in government after elections, which led to leadership turnover in state institutions. This, as indicated by the interviewees, was due to the power wielded by the Head of State in appointing directors of governmental institutions, sector ministers and Metropolitan, Municipal and District Chief Executives (MMDCE). Thus, their tenure of office is often linked with the incumbent government. Consequently, a change in government led to changes in leadership which usually affected coordination of already running programmes.

‘…. but they have this consultative group for different ministries … so, we had one for nutrition …. and we were meeting frequently and that’s how come we finalised the draft for the nutrition policy… and then the elections happened and then you know, that whole group resigned in mass so then there was a gap because of the turnover’. (Key informant – Academia)

### Funding for nutrition coordination

The issue of funding was a major gap within the whole implementation of the multi-sectoral approach. There was a unanimous assertion by the interviewees that insufficient funding was available for coordination. The interviews and learning journey data indicated that ministries often were unwilling to commit any of their resources towards collaborative works that do not emanate from them. Each institution therefore does not see the need to commit resources for another institution to manage, so once they are not much involved in the whole process, there would be little or no monetary commitment made as iterated by an interviewee from a government research facility.

‘If an institution is to coordinate a project, all other participating institution usually do not want to make any monetary commitment. Assuming I am in the Ministry of Agriculture and I have funds to implement my projects, and there is no allocation for coordination activity with Ministry of Health, everyone will be idle. So, I think when the budget is drawn, coordination activities should be included’. (Key informant – research institution)

The interview data highlighted the problem of ‘Who funds nutrition coordination’, as many of the stakeholders seemed uninterested to vote money for that purpose. Some of the interviewees thus called for the strengthening and funding of the Cross-sectoral Planning Group for Nutrition by government to lead in the coordination process.

### Power and governance within Ghana’s nutrition policy space

From the interview and document analysis data, power was identified at the national level within formal government institutions and structures. This power was visible in nature and largely demonstrated through policy formulation and implementation, and this was solely within the purview of government ministries. Data from the documentary analysis suggested an interesting interplay of power at the national level between the National Development Planning Commission (NDPC) and the MOH with regard to national nutrition policy formulation. While governance within the nutrition policy space was organised around the MOH (government’s policy-formulating body for health and nutrition issues), most of the interviewees noted that the NDPC was rather uniquely positioned to provide the needed leadership for nutrition through the Nutrition Cross-Sectoral Planning Group (NCSPG). For example, the MOH was lead in the formulation of the Ghana National Nutrition Policy but did not involve the NDPC as a collaborating stakeholder. It was evident from the interview data that policies emanating from the MOH were considered by some stakeholders as a monosectoral policy, rather than a comprehensive multi-sectoral national plan. This made it challenging for the MOH to oversee to the multi-sectoral nature of nutrition issues which were noted by a respondent from the local government ministry.

A common theme generated from the interview responses, especially from stakeholders at the subnational level, was their non-inclusion in policy development. Although most policies and programmes were to be implemented at the district level, their representation in the development of these policies was minimal, with the power space closed to them. Key informants from the subnational levels reported that they were usually brought on board only for training sessions after the policies have been formulated at the national level without their input.

‘For general national policies they will consult headquarters not the region, so if you are sitting at the region and districts, you may not have any idea until the policy comes out’. (Key informant –- regional health sector)

Interviewees indicated that international developmental partners such as the World Bank and the International Monetary Fund exerted both hidden and invisible forms of power on government policies due to their financial leverage. A stated example was the formulation of policies that led to trade liberalisation and the opening up of the local market for foreign goods. In contrast, organisations such as the WHO, FAO and the WFP were seen to wield visible and hidden power by running nutrition-related programmes and providing technical support as they augmented the efforts of government in addressing nutrition issues.

About one-third of the interviewees also identified big industry players – both local and foreign controlled – as having a covet influence on government policies through their relationships with the politicians, usually with a hidden agenda. These players financed the politicians in return for favours that drive their interests.

‘The politicians always have the most influence especially when policy involves the inflow of money. Those who bring in the money through the politicians seem to have the power’. (Key informant from academia)

The data from policy document reviews and the interviews showed that spaces for participation with respect to policy and programme design and development were usually closed to CSO. A classic example given was with the development of the national nutrition policy; there were no reported engagements with private sector industries and CSO. One interviewee noted that these CSO usually resort to advocacy, protests and boycotts in order to claim or create spaces for participation for themselves when they are initially excluded.

Key informants from the ministries of Agriculture and Trade and Industry indicated that they usually engaged with private industry actors during policy formulation and implementation. However, data from the learning journey and interviews with industry actors suggested differently; the spaces for participation were usually closed to them. For instance, reviewing the planting for food and jobs initiative, we noticed that the Ministry of Food and Agriculture was to link the policy to the One District, One Factory (1D1F) policy initiative via the public–private partnership in order to operationalise the 1D1F initiative. However, the interviewees from private industry discussed the lack of engagement by government during the policy formulation process as a key reason why the 1D1F initiative had yielded the expected results.

### Taking a look at governance under the Ghana AIDS Commission

Some interviewees discussed the great successes chalked in the fight against HIV/AIDS, with particular reference to the governance structure. Findings from the interview data and document review indicated the use of a multi-sectoral approach in addressing the HIV/AIDS pandemic through the establishment of the Ghana AIDS Commission (GAC), with the aim of providing leadership and coordinating the power dynamics involved in the national HIV/AIDS response.

‘I like what is being done on the issue of HIV…. the commission seems to be getting something right as they have been able to bring on board several key stakeholders to address the issues. We can all see the success they have achieved’. (Key informant from academia)

The established governance structure deployed by the GAC included a multi-sectoral governing board and a Steering Committee at the national level, with membership drawn from both public and private sectors and civil society in Ghana. In addition to the board having several sectoral ministers, the President of the Republic of Ghana was noted to be the chairperson of the governing board. This, as stated by some of the interviewees, gave the GAC the much-needed impetus to effectively operationalise its mandate. The commission also developed well-established structures at the subnational level, with the MMDCE tasked to provide the needed leadership at the subnational level, with set targets to meet. Documentary analysis revealed a positive effect in tackling the HIV/AIDS pandemic. Some interviewees also noted successful integration of the objectives of the GAC into the operations of all relevant sectors such as health, education, local government and rural development. They were also successful in bringing on board the chiefs, religious bodies and labour-related organisations.

‘The Ghana AIDS Commission campaign is successful because I believe they have good structures at the subnational level to drive the agenda from the national level. I worked with them a bit at the district level and they were able to bring us together to run successful campaigns’. (Key informants – district assembly and health)

## Discussion

Nutrition has long been identified as a multi-sectoral policy issue that requires a synergistic system to address arising issues^(12)^. Although the health sector often holds the relevant policy mandate for nutrition, sectors such as agriculture, and trade and industry, also play an important role in shaping policies and programmes that address the food environment and systems^(10,32,33)^. This study has found that though visible power (formal structures and responsibility) for nutrition was held by the health sector in Ghana, it has not been able to effectively coordinate a multi-sectoral approach to addressing all forms of malnutrition, especially overweight and obesity.

Also, power was identified at the national and subnational levels within formal government institutions and structures. Usually visible, this power was largely exercised through policy development and programme planning which mainly emanate from the national level and operationalised at the subnational levels (regional and districts assemblies). There was also weak coordination of the relevant sectors and stakeholders within the policy space of nutrition. Coordination fell outside the scope of the MOH as the NDPC was identified by the interviewees as uniquely placed to drive the multi-sectoral nutrition agenda. Efforts were targeted at the national level with no well-organised formal operational structures at the subnational levels; thus, actors at those levels felt marginalised as the spaces for participation remained closed to them.

Again, the turnover of leaders at the top of institutions created challenges in the continuity of nutrition programmes. Divorcing the leadership from political influences or having a fixed tenure of office for these leaders could prove effective in addressing the turnover issues. Even though the president appoints Justices to the supreme court, or the Commissioner of the Electoral Commission, these appointees are immune from regular political turnovers. Thus, the top hierarchy of the NCSPG could benefit from such arrangements.

### Nutrition governance and power: a lesson from the Ghana AIDS Commission

Reflecting on our findings, there is an evident parallel with the response adopted to curb the HIV/AIDS pandemic in Ghana, where a whole of system approach was put in place to address the intricacies of HIV/AIDS management and prevention. HIV/AIDS was considered not only as a medical problem in Ghana but also a developmental issue, and thus, spaces for participation were created at all levels of governance to summon all the systems for development to curtail its spread, with formal operational structures at the subnational levels as well^(32,34)^. Just like in the case of the GAC, elevating the NCSPG to the status of a National Nutrition Council with the backing of the office of the president, as well as creating a multi-stakeholder platform at the regional, district and assembly levels could help create the needed spaces for participation by the grassroots to aid in addressing nutrition issues.

### Addressing the issue of funding

The identified gap of financing collaborative programmes was usually due to the fact that there are no budget lines for coordination and collaboration programmes and activities, therefore making it difficult to fund such initiatives^(35,36)^. Among other revenue sources, taking a look at taxation of sugar and SSB could provide an avenue to generate funds to address the financing gap. It was evident that prices of some SSB are so low that slogans like ‘still Gh1 cedi p3’, implying that the price of a 300 mls of that particular carbonated SSB only costs Gh1 cedi (about US $ 0·17) as at 2020, have been running for over 3 years. These low prices of SSB have been linked to increased consumption which has resulted in the increase in obesity and overweight prevalence^(37,38)^. An introduction of sugar tax on SSB, however, could help to address this nutrition-related issue^(39)^ while raising revenue to tackle malnutrition. South Africa, amidst pressures from many stakeholders – particularly the SSB industry, finally followed the footsteps of countries like Hungry, Finland, Mexico and the UK in passing a tax on SSB, the Health Promotion Levy. It imposed a 0·021ZAR tax for every gram of sugar content that surpasses 4 g/100 ml^(40)^. Ghana can explore such an option to help fund interventions directed at reducing obesity and diet-related non-communicable diseases.

The strength of this study is in its ability to deal with a variety of evidence (documents, interviews and learning journey discussions) as data sources^(31)^. In particular, the learning journey provided a shared perspective to enhance the participants’ discussion^(30)^. However, one limitation was that much of the analysis of power needed to be inferred from the participant interviews, as it was challenging to ask about power directly. In the documents analysis, priority was given to documents produced within the past decade (2020–2010), as such some related documents before the past decade may have been missed. We recommend further review of the 2012 and 2022 National NCD Policy documents to ascertain any improvements in tackling obesity and diet-related non-communicable diseases in Ghana.

## Conclusion

In addressing the multi-faceted issues of nutrition, efforts from several actors in the planning and implementation of policies are needed. There is the need for well-organised structures at both the national and subnational levels to drive the needed change. Thus, reorganising the NCSPG into a National Nutrition Council, with the backing of the Office of the President, could be more effective in managing the power dynamics. Consideration of taxation on SSB could provide some needed funding to tackle the coordination of nutrition programmes addressing the issue of obesity.
